# Characteristics of endophytic bacteria and active ingredients in the Eucommiae cortex from different origins

**DOI:** 10.3389/fmicb.2023.1164674

**Published:** 2023-05-17

**Authors:** Xuejuan Liang, Kang Zhou, Penghui Li, Dan Wan, Jing Liu, Xin Yi, Yanmei Peng

**Affiliations:** ^1^Innovative Medicine Institute of Traditional Chinese Medicine, Hunan Academy of Chinese Medicine, Changsha, China; ^2^College of Pharmacy, Hunan University of Chinese Medicine, Changsha, China; ^3^College of Chinese Medicine, Hunan University of Chinese Medicine, Changsha, China

**Keywords:** *E. ulmoides*, Eucommiae cortex, endophytic bacteria, origin, active ingredient

## Abstract

**Objective:**

This study aimed to explore the differences between Eucommiae cortex (EC) endophytic bacteria from different origins and their effects on the active ingredients of EC.

**Methods:**

A total of 10 samples of *Eucommia ulmoides* Oliv. (*E. ulmoides*) bark were collected from each of the following four regions, namely, Zunyi in Guizhou (GZ), Baokang in Hubei (HUB), Cili in Hunan (HUN), and Loyang in Shaanxi (SX). Subsequently, the contents of the main active ingredients of EC were determined by ultra-performance liquid chromatography (UPLC), and the endophytic bacteria of EC were detected by 16S rRNA sequencing. The relationship between the dominant endophytic bacteria and the active ingredients was investigated by correlation analysis.

**Results:**

A total of 4,551 different operational taxonomic units (OTUs) were delineated in the four groups of samples, of which 585, 439, 957, and 684 genera were annotated from GZ, HUB, HUN, and SX, respectively. The richness and diversity of endophytic bacteria from different origins were ranked as HUN > SX > GZ or HUB. The analysis demonstrated that there was no significant correlation between the diversity and richness of endophytic bacteria in EC and its active ingredients. Nevertheless, notable variations in the community structures of endophytic bacteria were observed across different origins, and they had a considerable impact on certain active ingredients in EC. *Comamonas* and *Cedecea* were the dominant genera. Characteristic bacteria of different origins could be clearly distinguished. Simultaneous, significant correlations had been identified between some characteristic endophytic bacteria derived from different origins and active ingredients of EC. For example, *Delftia*, a characteristic bacterium from GZ, showed a significant positive correlation with pinoresinol diglucoside. *Paenibacillus* and *Klebsiella*, two characteristic bacteria from HUB, exhibited significant positive correlations with geniposidic acid. *Thauera*, a characteristic bacterium from HUN, demonstrated a significant positive correlation with geniposide. *Brevundimonas*, a characteristic bacterium from SX, displayed a significant positive correlation with pinoresinol diglucoside.

**Conclusion:**

There was a complex correlation between EC endophytic bacteria and active ingredient content, while EC endophytic bacteria from different origins had significant differences at the genus level.

## 1. Introduction

*E. ulmoides* belongs to the deciduous tree of Eucommiaceae and *Eucommia*, mainly distributed in Guizhou, Hunan, Shaanxi, and Hubei provinces, south of the Qinling mountains. The cortex of *E. ulmoides* is a drug used in traditional Chinese medicine (TCM), commonly used to treat osteoporosis, soreness of the waist and knees, dizziness, fetal movement disturbance, and other symptoms, and has a medical history of nearly 2000 years (Feng et al., 2016). Modern studies have shown that iridoids (including aucubin, geniposide, and geniposidic acid) and lignans (including pinoresinol diglucoside and syringaresinol diglucoside) are the main active ingredients of EC (Hussain et al., 2016; Huang et al., 2021). Among them, aucubin and geniposide have anti-diabetic, anti-osteoporosis, anti-hypertension, and anti-inflammatory biological activities (Zeng et al., 2020), while pinoresinol diglucoside, geniposide, and geniposidic acid can promote osteoblast proliferation and inhibit osteoclast activity (Ha et al., 2003). Geniposidic acid and syringaresinol diglucoside are pharmacodynamic ingredients exerting the antihypertensive effect of EC (He et al., 2014; Ishimitsu et al., 2021).

The contents of active ingredients in medicinal plants are closely related to their unique ecological environment, which includes not only abiotic factors, such as light, climate, and temperature, but also the microbial ecology with plants (Yang J. et al., 2019). Symbiotic endophytes widely inhabit plant tissues without causing obvious abnormal host conditions. Many studies have shown that endophytes play vital roles in plant growth, development, disease control, and stress resistance (Köberl et al., 2013; Liu et al., 2022; Pathak et al., 2022). Meanwhile, certain endophytes can also promote the synthesis of active metabolites in medicinal plants by producing exogenous inducers or by producing similar metabolites in the host through the related synthesis pathway, thereby facilitating the accumulation of medicinal ingredients in plants (Zhong et al., 2016; Rustamova et al., 2020). For example, Song et al. (2017) reported that the endophytic bacterium LB 5-3 of Radix Ginseng could promote the accumulation of ginsenoside in the adventitious root cultures. Liu et al. (2020) reported that the dominant endophytic bacteria *Microbacterium* and *norank_f_7B-8* in *Coptidis rhizome* had significant correlations in the synthesis of berberine. In terms of EC, Liu et al. (2011) isolated three strains of fungi with the effect of producing pinoresinol diglucoside among the EC, and the one with the highest yield was identified as *Phomopsi*s sp. The authors' further research (Shi et al., 2012) also certified that the microbial PDG by fermentation was the same as plant-derived PDG. Moreover, the contents of active ingredients and the community diversity of endophytes in different ecological environments EC also differ (Cai et al., 2014; Liang et al., 2014). A notable example is Yan et al. (2017) examining EC ingredients from Meitan in Guizhou, Lichuan in Hubei, and Ningqiang in Shaanxi. They found that the active ingredients of EC ingredients from different origins could be distinguished, and there were different patterns of variation in 14 common ingredients. In our previous report (Liang et al., 2014), the EC endophytic fungal composition of three origins, GZ, HUN, and SX, were significantly different. However, the majority of the current research studies on EC endophytes are focused on fungi, with a limited investigation into bacteria as endophytes in EC, and the existing studies primarily targeted the detection of culturable endophytic bacteria (Zhao et al., 2016).

In addition, TCM focuses on the geographic location of high-quality herbs, i.e., “Daodi.” However, the current wild resources of *E. ulmoides* are scarce, the boundaries of Daodi are blurred, the structure of endophytic bacteria of *E. ulmoides* is unclear, and the quality of herbs varies. The establishment of quality evaluation and the Daodi identification method of EC could help to clarify the Daodi production area of EC and improve the quality of herbs. Therefore, this study analyzed the characteristics of endophytic bacteria in EC from GZ, HUN, SX, and HUB by 16S rRNA sequencing technology and performed correlation analysis with their active ingredients in order to explore the distribution characteristics of endophytic bacteria in high-quality EC. The results can provide an experimental basis for clarifying the role of characteristic bacteria in forming EC quality and screening characteristic bacteria of the origins.

## 2. Material and methods

### 2.1. Sample collection

The fresh cortex of *E. ulmoides* was collected from Zunyi in Guizhou (GZ), Cili in Hunan (HUN), Loyang in Shaanxi (SX), and Baokang in Hubei (HUB), respectively. The sample collection information is shown in Table 1. In total, 40 healthy *E. ulmoides* trees above 6 years old were randomly selected in different sampling areas. At 1–1.5 m above the ground, fresh bark samples were peeled from each tree, immediately put in a sterile plastic bag, and transported to the laboratory within 48 h at 4°C.

**Table 1:** Sample collection information.

**Sample number**	**Sample size**	**Origins**	**Acquisition time**
GZ1 to GZ10	10	Zunyi county, Guizhou province	2021.5.7
HUB1 to HUB10	10	Baokang county, Hubei province	2021.5.24
HUN1 to HUN10	10	Cili county, Hunan province	2021.4.28
SX1 to SX10	10	Loyang county, Shaanxi province	2021.5.9

### 2.2. Sample processing

The collected EC was split into two parts. One was microwave-dried for 3 min, dried at 50°C, and stored for the detection of active ingredients. The other sample was cut into small pieces of ~3 cm × 3 cm with a sterile knife, rinsed with sterile water for 30 s, blotted with a sterile filter paper, soaked in 75% ethanol for 2 min and in 5.5% sodium hypochlorite solution for 3 min, then rinsed with 75% ethanol for 30 s, washed with sterile water three times, and blotted with the sterile filter paper. Finally, the phellem layer was scraped off, and the tissue pieces of the phloem of ~0.4 cm^2^ in the center were taken. After uniformly mixed, the tissue pieces were stored in 10 ml sterile centrifuge tubes at −80°C for DNA extraction of entophytic microbiota. The sample DNA extraction, amplification, and library sequencing were provided by Majorbio Bio-Pharm Technology Co. Ltd. (Shanghai, China).

### 2.3. Active ingredient detection

#### 2.3.1. Chemicals and reagents

Five reference compounds, including aucubin, geniposidic acid, geniposide, pinoresinol diglucoside, and syringaresinol diglucoside, were purchased from Chengdu Chroma-Biotechnology Co., Ltd. (Chengdu, China). The purities of all the above reference substances were more than 98% as determined by high-performance liquid chromatography (HPLC) analysis. HPLC-grade formic acid, methanol, and acetonitrile were purchased from Merck (Darmstadt, Germany).

#### 2.3.2. Preparation of sample extract

After the cork was removed, the phloem of the dried EC was cut, and then kneaded into floccules. In total, 2 g of the floccules was added to 30 ml of 50% methanol, weighed, and then treated with ultrasound (500 W, 40 kHz) for 30 min at room temperature. It was weighed again after cooling, and 50% methanol was used to make up for the loss of weight. The filtrate was obtained by filtration using a 0.22 μm Millipore filter unit for future UPLC analysis.

#### 2.3.3. UPLC analysis

The analysis of EC active ingredient contents of different origins using the UPLC method with the Agilent 1290 ultra-performance liquid chromatography (Agilent Technologies Inc, USA). Chromatographic column: waters ACQUITY UPLC^®^ BEH C_18_ 1.7 μm (2.1 × 100 mm), column temperature: 35°C, flow rate: 0.3 ml/min, detection wavelength: 208 nm, 240 nm, injection volume: 2 μl, mobile phase system: acetonitrile (A) −0.1% formic acid water (B), gradient elution (mobile phase): 0–4 min, 3–7% A; 4–10 min, 7–3% A; 0–11 min, 3–10% A; 11–25 min, 10–14% A; 25–32 min, 14–30% A; 32–34 min, 30% A; 32.01–39 min, 3% A.

#### 2.3.4. Methodology validation

The five reference standards were weighed accurately and dissolved with methanol comparable to at least six appropriate concentrations of each compound. The precision was determined by analyzing the sample solution six consecutive times. Stability was evaluated by calculating the relative standard deviation (RSD) of signal intensity of the same tested solution at 0, 2, 4, 8, 12, 24, and 36 h. Recovery experiments were done by spiking authentic standard solutions into samples directly.

### 2.4. DNA extraction and PCR amplification

After surface disinfection, the DNA extraction kit (Omega Mag-Bind soil DNA kit, Norcross, GA, USA) was used to extract total microbial genomic DNA from EC samples according to the manufacturer's instructions. The quality and concentration of DNA were evaluated using 1.0% agarose gel electrophoresis and a NanoDrop^®^ ND-2000 spectrophotometer (Thermo Scientific Inc., USA) and were then stored at −80°C until further use.

PCR amplification was performed on DNA that met the quality requirements. Amplification system (20 μL) comprised 5 × FastPfu Buffer 4 μl, dNTP (2.5 mM) 2 μl, forward primer (5 uM) 0.8 μl, reverse primer (5 uM) 0.8 μl, FastPfu Polymerase 0.4 μl, BSA 0.2 μl, and DNA Template 10 ng, supplemented with ddH_2_O to 20 μl. Amplification parameters were as follows: initial denaturation at 95°C for 3 min, followed by denaturing at 95°C for 30 s, annealing at 55°C for 30 s, extension at 72°C for 45 s, final extension at 72°C for 10 min, and 10°C hold, repeated for 13–15 cycles. To reduce the contamination of chloroplast DNA in plants, endophytic bacteria were sequenced using 16S V5–V7 region primers (forward primer: 799F, AACMGGATTAGATACCCKG, reverse primer: 1193R, ACGTCATCCCCACCTTCC) (Wang et al., 2018). All samples were amplified in triplicate. The PCR products were extracted from 2% agarose gel and purified using the AxyPrep DNA Gel Extraction Kit (Axygen Biosciences, Union City, CA, USA) according to the manufacturer's instructions and quantified using the Quantus™ Fluorometer (Promega, USA). Purified amplicons were pooled in equimolar amounts and paired-end sequenced on an Illumina NovaSeq PE250 platform (Illumina, San Diego, USA) according to the standard protocols by Majorbio Bio-Pharm Technology Co. Ltd.

### 2.5. Bioinformatics analysis

The obtained data were processed using Flash (v 1.2.11) sequence splicing, QIIME (v 1.9.1) (Caporaso et al., 2010) to generate abundance tables for each classification level, Uparse (v 11) clustering, and RDP classifier (v 2.13) classification annotation. The optimized sequences were clustered into operational taxonomic units (OTUs) with a 97% sequence similarity level. The OTU table was manually filtered, i.e., chloroplast and mitochondria sequences in all samples were removed. The taxonomy of each OTU representative sequence was analyzed by the RDP classifier against the 16S rRNA gene database (Silva v138/16s_bacteria) using a confidence threshold of 0.7. The OTU abundance dataset was normalized using a standard sequence number corresponding to the sample with the least sequences. All subsequent analyses were performed based on the normalized data. The number of unique or common OTUs among different groups was represented using a Venn diagram. Shannon curves were used to assess whether the sequencing depth of the samples met the criteria, and Mothur (v 1.30.2) (Schloss et al., 2009) was used to calculate ACE, Chao, Shannon, and Simpson indexes to assess the alpha diversity of endophytic bacterial communities of different origins of EC (Liu et al., 2021). Among them, ACE and Chao indices were positively correlated with species richness. The Shannon index was positively correlated with species diversity. A scatter plot showing the correlation between alpha diversity and active ingredients in EC was generated using Spearman's rank correlation coefficient.

The beta diversity of endophytic bacterial communities in EC samples of different origins was evaluated using the principal coordinate analysis (PCoA) and hierarchical clustering analysis based on the Bray–Curtis distance. The differences in community composition were tested using Adonis. Redundancy analysis (RDA) was used to reflect the relationship between active ingredients of EC and the distribution of endophytic bacterial communities. Linear discriminant analysis effect size (LEfSe) was used to screen the marker difference species in each group of samples. The correlation analysis between the endophytic bacterial community and the content of active ingredients was performed using Spearman's correlation coefficient method, and heatmaps were drawn using R (v 4.2.2).

### 2.6. Statistical analysis

SPSS 21.0 software was used for statistical analysis. Experimental data were expressed as mean ± standard deviation. The ANOVA test was used if the normal distribution and homogeneity of variance were met between multiple groups; otherwise, the Kruskal–Wallis rank sum test was used. A *P*-value of <0.05 indicates that the difference is statistically significant.

## 3. Results

### 3.1. EC active ingredient content of different origins

The performance validation of the method was evaluated, including linearity, precision, reproducibility, stability, and recovery (Table 2). Pinoresinol diglucoside, aucubin, geniposidic acid, geniposide, and syringaresinol diglucoside all show good linearity (*R* ≥ 0.999). The five active ingredients' RSD of precision, repeatability, stability, and recovery were ≤3%. This indicates that the UPLC method was satisfactory for subsequent analysis of all EC samples in this study.

**Table 2:** Liner equations, precision, stability, repeatability, and recovery of quantification of five compounds.

**Compounds**	**Liner equations**	***R* ^2^**	**Linearity range/μg**	**Precision RSD (*n* = 6)**	**Stability RSD (*n* = 6)**	**Repeatability RSD (*n* = 6)**	**Recovery**	**Recovery RSD**
Aucubin	y = 2633.2x + 780.58	0.9996	0.214–10.64	0.47%	1.65%	1.96%	97.94%	2.89%
Geniposidic acid	y = 3467.6x + 61.569	0.9991	0.043–10.68	0.86%	1.04%	2.45%	103.58%	1.98%
Geniposide	y = 8674.7x + 70.569	0.9995	0.004–2.256	0.35%	1.58%	2.68%	98.38%	2.47%
Pinoresinol diglucoside	y = 18485x + 361.21	0.9990	0.042–2.124	0.80%	2.20%	2.08%	97.22%	2.39%
Syringaresinol diglucoside	y = 7682.7x + 1.0254	0.9988	0.076–1.520	1.29%	2.63%	2.69%	95.91%	2.92%

**Figure 1:**
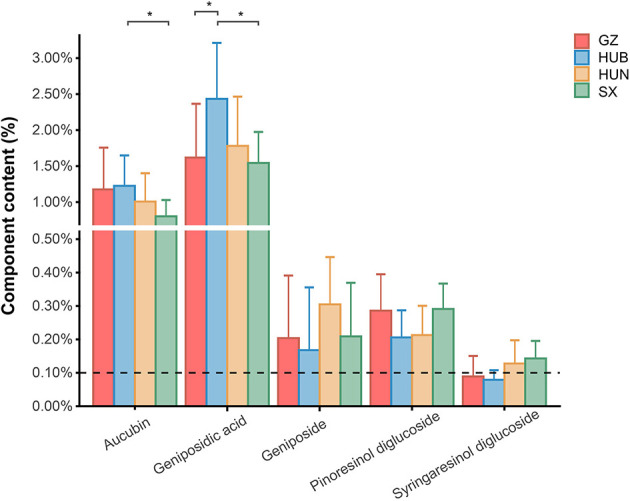
Content of active ingredients in EC from different origins. GZ, Zunyi in Guizhou; HUB, Baokang in Hubei; HUN, Cili in Hunan; SX, Loyang in Shaanxi. *P < 0.05.

The contents of pinoresinol diglucoside, aucubin, geniposidic acid, geniposide, and syringaresinol diglucoside in EC from GZ, HUB, HUN, and SX are shown in Figure 1. The Chinese Pharmacopeia (2020 version) stipulates that the content of pinoresinol diglucoside in EC should not be <0.10%. In the pinoresinol diglucoside content, the four EC origins are ranked as SX = GZ > HUN = HUB, the highest was 0.30% and the lowest was 0.21%, all of which met the pharmacopeia requirements. In the aucubin content, the four EC origins are ranked as HUB > GZ > HUN > SX, where the HUB was significantly higher than that of SX (*P* < 0.05). In the geniposidic acid content, the four EC origins are ranked as HUB > HUN > GZ > SX, where the HUB was significantly higher than that in GZ and SX (*P* < 0.05), reaching 2.55%. In the geniposide content, the four EC origins are ranked as HUN > SX > GZ > HUB. In the syringaresinol diglucoside content, the four EC origins are ranked as SX > HUN > GZ > HUB. These indicate different patterns of variation in the content of active ingredients among different origins.

### 3.2. Alpha diversity of endophytic bacteria of EC from different origins

**Figure 2:**
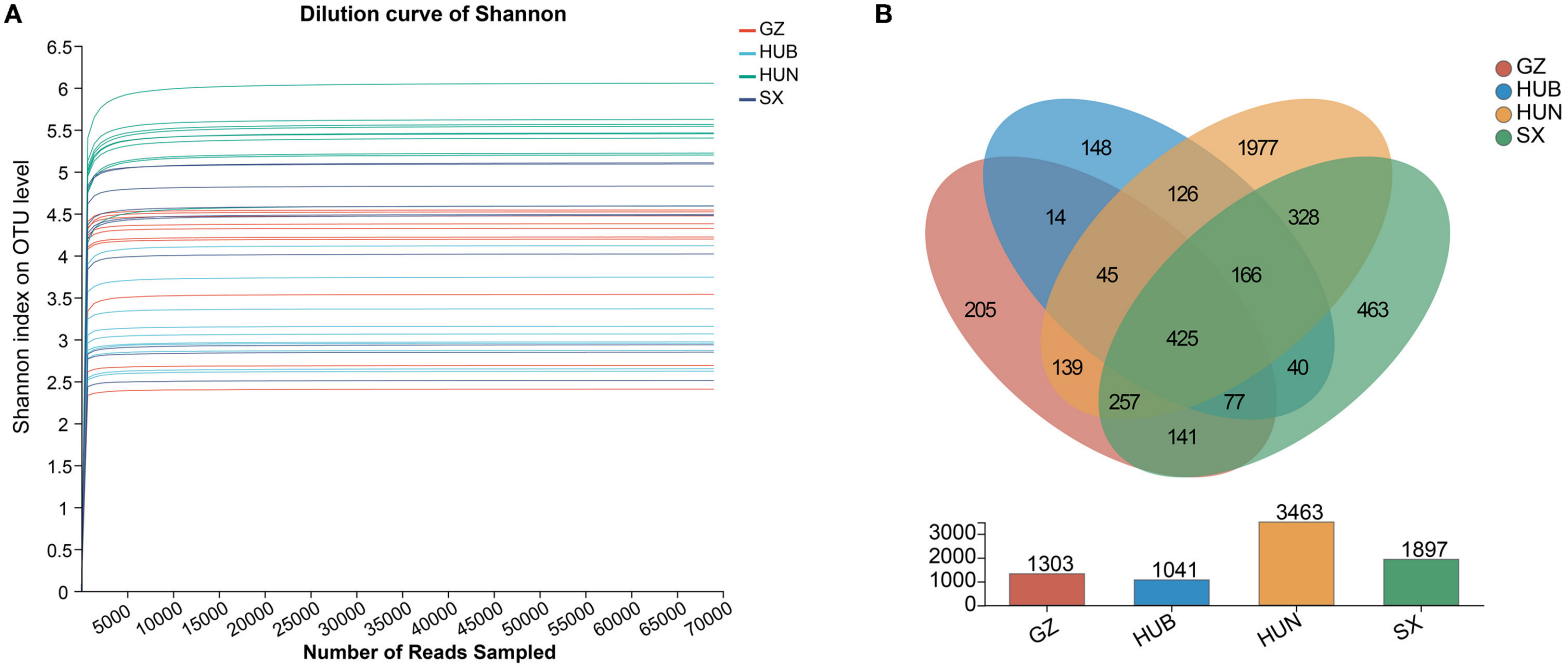
Venn diagram and dilution curve of endophytic bacteria OTUs in EC from different origins. **(A)** Venn diagram. **(B)** Dilution curve of Shannon. GZ, Zunyi in Guizhou; HUB, Baokang in Hubei; HUN, Cili in Hunan; SX, Loyang in Shaanxi.

As shown in Figure 2A, the curve for each sample flattens out when the sequenced fragment reaches 5000, and the gain of increasing the number of sequencings on the number of OTUs is no longer significant. This indicates that the current sequencing quantity is sufficient to reflect most microbial information in the samples. As shown in Figure 2B, the number of OTUs shared by the four origins was 425. Among them, the number of OTUs from HUN was the most (*n* = 3,463), followed by SX (n = 1,897), GZ (*n* = 1,303), and HUB (*n* = 1,041). The numbers of OTUs unique to GZ, HUB, HUN, and SX samples were 205, 148, 1,977, and 463, respectively. Therefore, the results tentatively suggest that EC endophytic bacterial richness was highest in the HUN sample.

**Figure 3:**
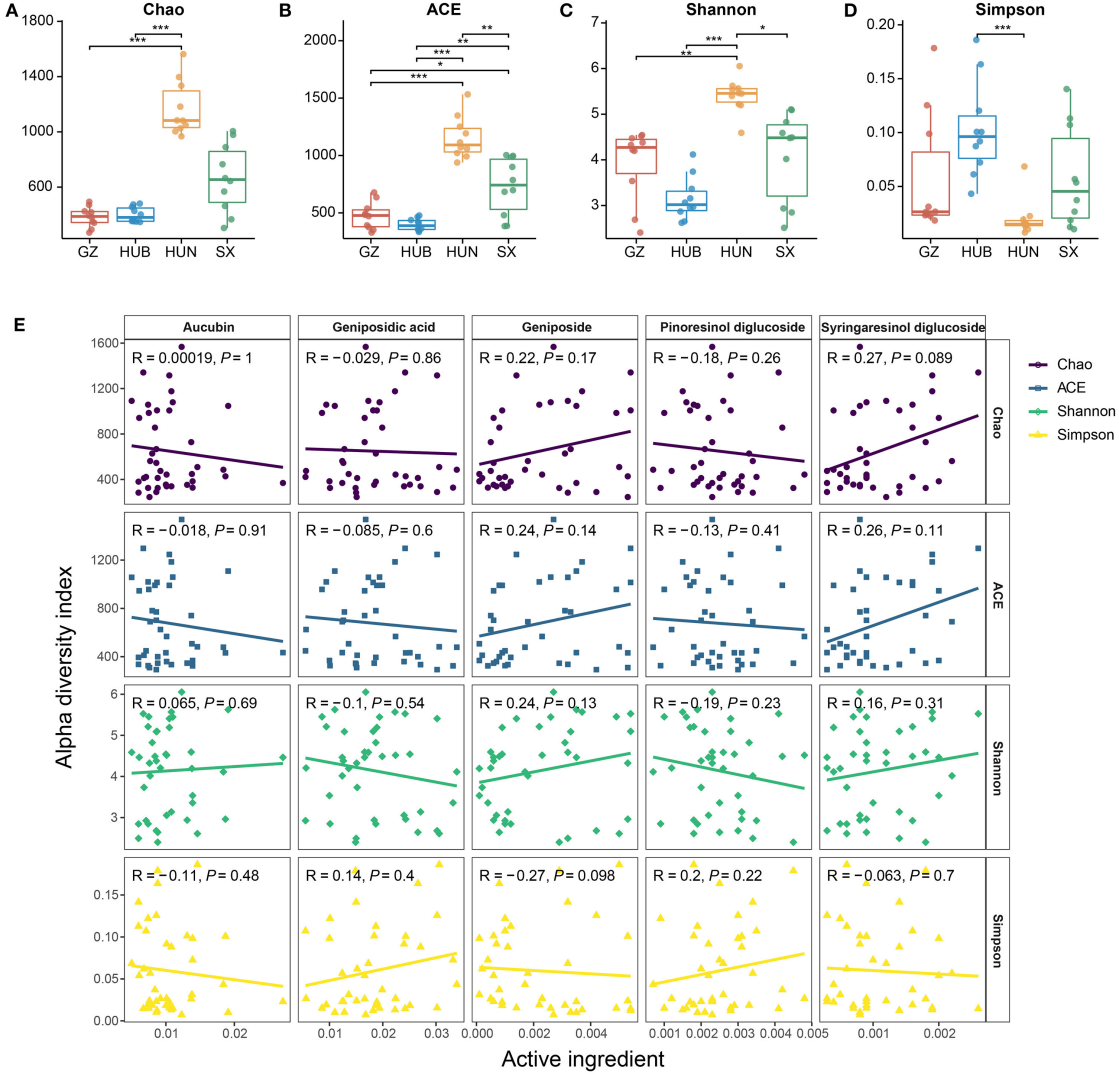
Alpha diversity of endophytic bacteria in EC from different origins. **(A)** Chao. **(B)** ACE. **(C)** Shannon. **(D)** Simpson. **(E)** Scatter diagram of a correlation between the alpha diversity index and active ingredients. GZ, Zunyi in Guizhou; HUB, Baokang in Hubei; HUN, Cili in Hunan; SX, Loyang in Shaanxi. *P < 0.05, **P < 0.01, and ***P < 0.001.

From the Chao and ACE indices in Figures 3A, B, it can be seen that the endophytic bacterial richness of EC in HUN was significantly higher than other origins (*P* < 0.01 or p < 0.001), SX ranked the second in richness, and HUB or GZ had the lowest richness. The Shannon index (Figure 3C) showed HUN > SX > GZ > HUB, where HUN was significantly higher than SX (*P* < 0.05), GZ (*P* < 0.01), and HUB (*P* < 0.001) samples, which was consistent with the OTU results. The Simpson index (Figure 3D) showed HUN < GZ < SX < HUB, where HUB was significantly higher than the HUN (*P* < 0.001). This shows that the richness and diversity of EC endophytic bacteria from HUN were the highest, while those from GZ and HUB were the lowest. The results of the correlation between the active ingredients and the alpha diversity index of EC are shown in Figure 3E (Spearman correlation coefficient). There was no significant correlation between the five active ingredients and the four alpha diversity indices (*R*^2^ < 0.3, *P* > 0.05).

### 3.3. Beta diversity of endophytic bacteria of EC from different origins

**Figure 4:**
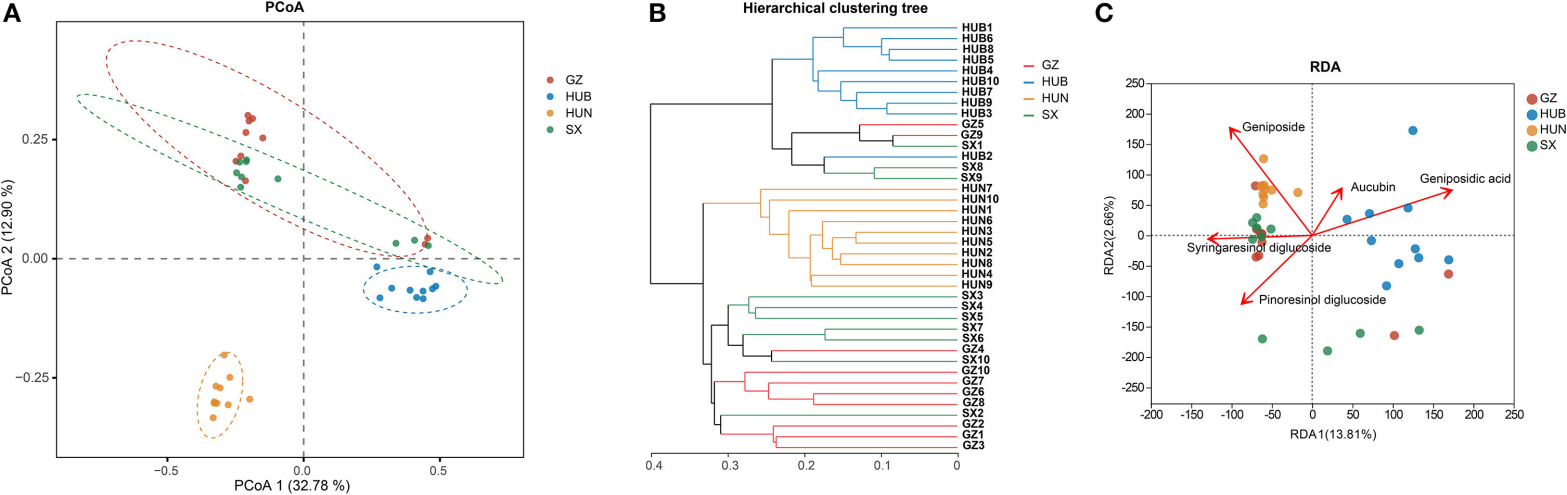
Beta diversity of endophytic bacteria in EC from different origins. **(A)** PCoA (based on Bray–Curtis distance). The closer the two points in the diagram were, the more similar the structure of the sample community representing the two points would be. **(B)** Hierarchical clustering tree (based on Bray–Curtis distance). **(C)** RDA of active ingredients and endophytic bacterial community in EC. GZ1-10, Zunyi in Guizhou 1-10; HUB1-10, Baokang in Hubei 1-10; HUN1-10, Cili in Hunan 1-10; SX1-10, Loyang in Shaanxi 1-10.

As shown in Figure 4A, the contributions of PC1 and PC2 were 32.78 and 12.90%, respectively. The samples from HUN and HUB were distributed in the third and fourth quadrants, respectively, and were significantly separated from the bacterial communities of other origins (*P* < 0.01), while GZ and SX were similarly distributed and the differences in the bacterial communities were not significant (*P* > 0.05). This indicates that the community structure of the EC endophytic bacteria from SX and GZ is more similar. Compared with other origins, the community structure of EC endophytic bacteria from HUB and HUN were different, with the most obvious difference in HUN. In the clustering analysis (Figure 4B), samples from HUN were the least distant from each other and could be well-clustered into one group. The samples from HUB also cluster well-together except for the HUB2, while the samples from GZ and SX are relatively scattered. This indicates that the intra-group variation of EC endophytic bacteria from HUN was the smallest, followed by that in HUB, and the intra-group variation of EC endophytic bacteria from SX and GZ was relatively large. According to the RDA results, the community structure of endophytic bacteria was significantly correlated with geniposidic acid, geniposide, and pinoresinol diglucoside (*P* < 0.001, *P* < 0.01, *P* < 0.05) (Table 3). The content of geniposidic acid mainly affected the EC endophytic bacterial community from HUB, the content of geniposide mainly affected the EC endophytic bacterial community from HUN, and the content of pinoresinol diglucoside mainly affected the EC endophytic bacterial community from SX and GZ (Figure 4C).

**Table 3:** RDA results.

**Compounds**	***R* ^2^**	***P*-values**	**Mark**
Aucubin	0.0626	0.324	
Geniposidic acid	0.3128	0.001	***
Geniposide	0.2098	0.009	**
Pinoresinol diglucoside	0.1915	0.019	*
Syringaresinol diglucoside	0.1169	0.096	

^*^P < 0.05, ^**^P < 0.01, and ^***^P < 0.001.

### 3.4. The taxonomic composition of endophytic bacteria of EC from different origins

**Figure 5:**
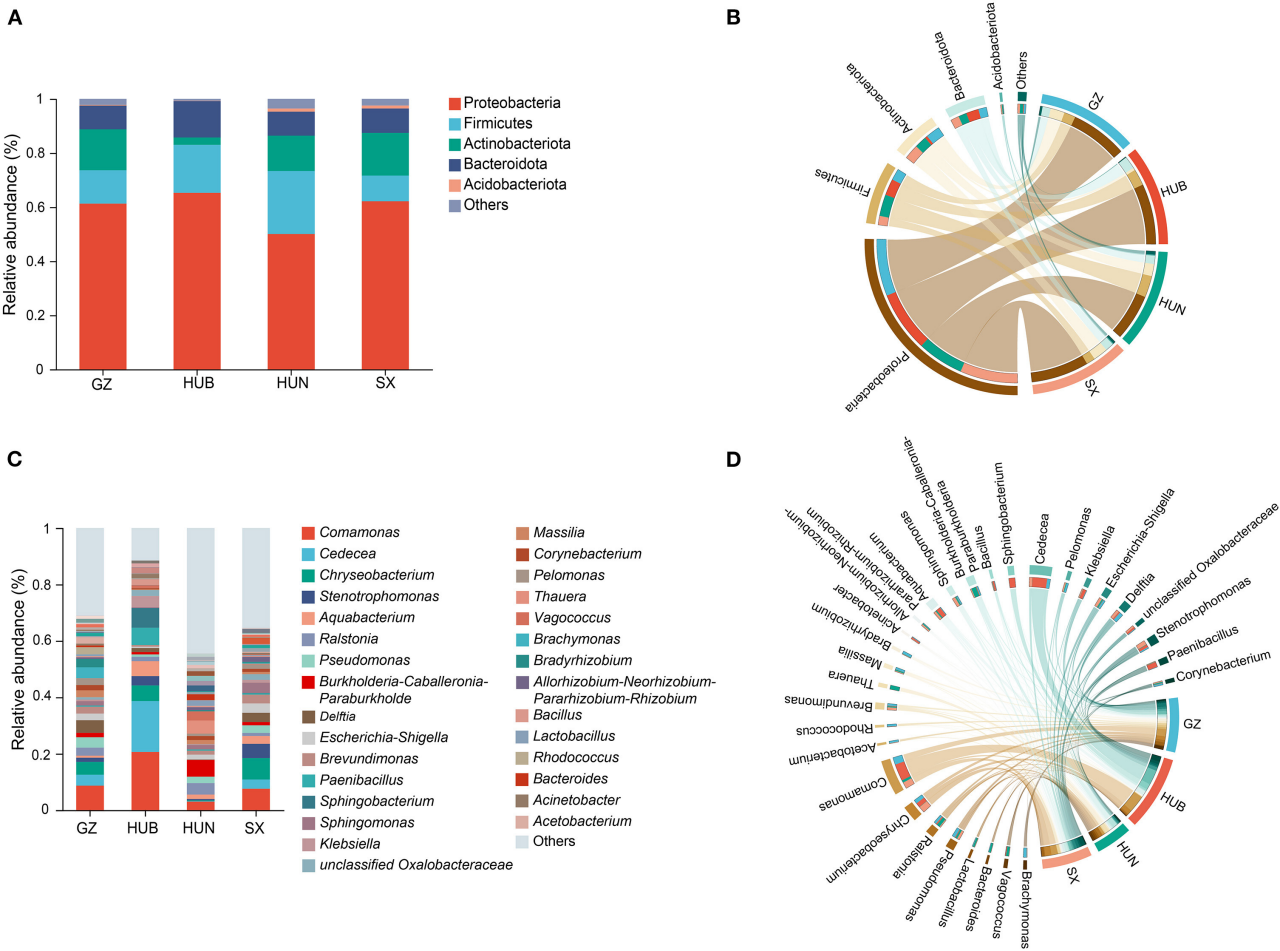
Taxonomic composition of endophytic bacteria in EC of different origins. **(A)** Histogram of relative abundance at the phylum level. **(B)** Chord diagram of the phylum level. **(C)** Histogram of relative abundance at the genus level. **(D)** Chord diagram of the genus level. GZ, Zunyi in Guizhou; HUB, Baokang in Hubei; HUN, Cili in Hunan; SX, Loyang in Shaanxi.

The endophytic bacteria of EC from these four origins were analyzed at the phylum and genus levels based on the OTU species annotation. At the phylum level (Figures 5A, B), the endophytic bacteria of EC from the four origins consisted of Proteobacteria (50.06–65.19%), Firmicutes (9.53–23.33%), Actinobacteriota (2.68–15.77%), Bacteroidota (8.75–13.51%), and Acidobacteriota (0.13–1.15%), and low abundance taxonomic clades accounted for <1% of the total. At the genus level (Figures 5C, D), 585, 439, 957, and 684 genera were detected in samples from GZ, HUB, HUN, and SX, respectively. Among them, HUN had the largest number of genera and HUB had the least. Genera with a relative abundance percentage >1% were selected for graphical display (Figures 5C, D). At the genus level, some dominant bacteria in EC show considerable differences in abundance. For instance, the relative abundance of *Comamonas* of EC from HUB is 20.71%, while that from HUN is only 3.02%. Similarly, the relative abundance of *Chryseobacterium* of EC from SX is 7.61%, while that from HUN is only 0.28%. In addition, the dominant bacteria of EC also vary across different origins. For example, the top three endophytic bacteria with abundance in EC from HUB are *Comamonas* (20.71%), *Cedecea* (18.08%), and *Sphingobacterium* (7.00%). On the other hand, the top three endophytic bacteria with abundance in EC from HUN are *Burkholderia-Caballeronia-Paraburkholderia* (5.92%), *Thauera* (4.64%), and *Ralstonia* (4.07%).

### 3.5. Endophytic bacteria with EC characteristics of different origins

**Figure 6:**
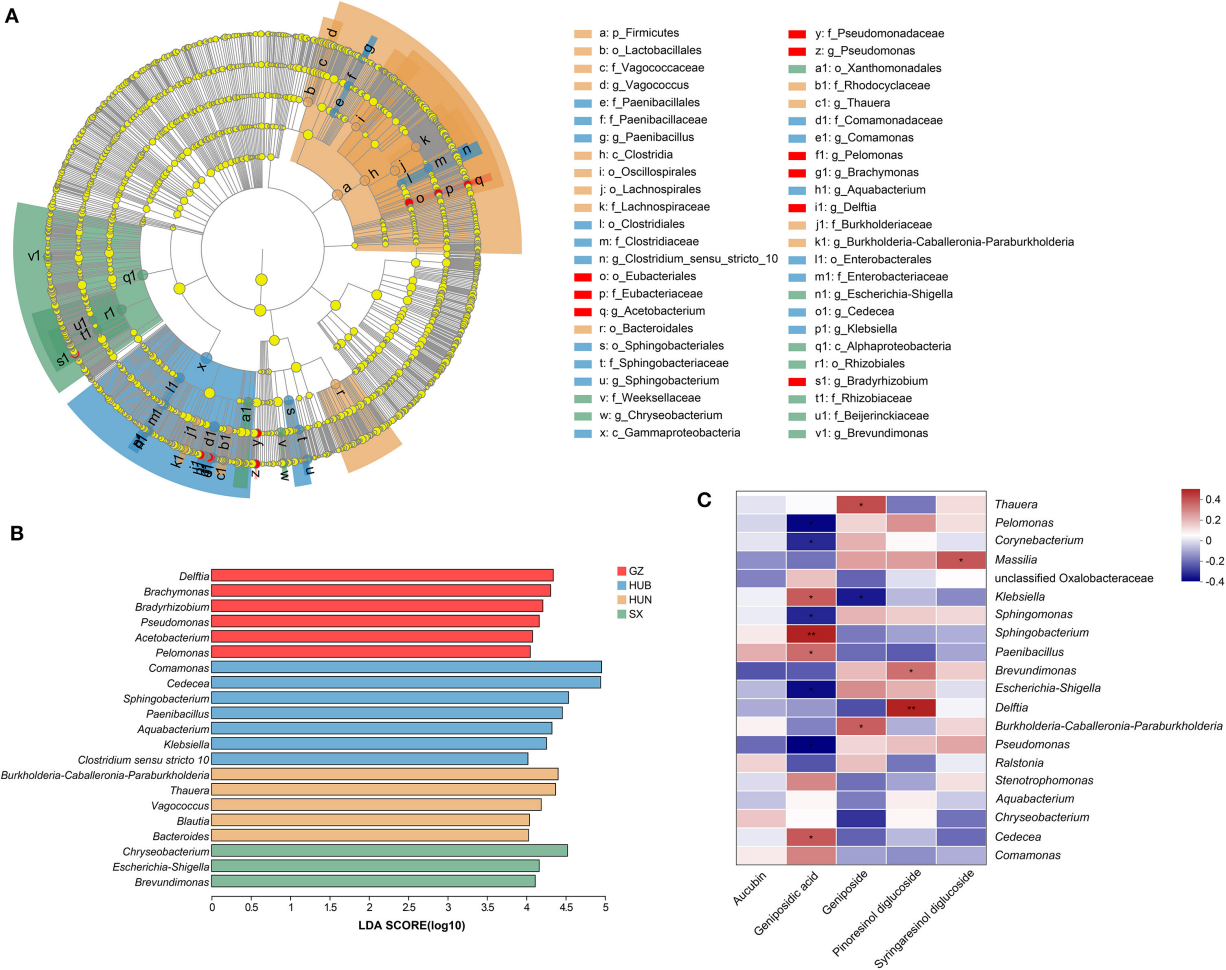
Characteristic endophytic bacteria of EC from different origins and correlation analysis between dominant endophytic bacteria of EC with active ingredients. **(A)** Cladogram diagram. **(B)** LDA score diagram. **(C)** Correlation heatmap between dominant endophytic bacteria and active ingredients in EC. In the diagram, red indicates a positive correlation, blue indicates a negative correlation, and a darker color indicates a stronger correlation. *P < 0.05, **P < 0.01. GZ, Zunyi in Guizhou; HUB, Baokang in Hubei; HUN, Cili in Hunan; SX, Loyang in Shaanxi.

A Lefse analysis was conducted to screen out characteristic endophytic bacteria in EC samples from different origins. Based on the linear discriminant analysis (LDA) with a threshold of ≥4, this study obtained 48 characteristic taxa and 21 characteristic genera, as shown in Figures 6A, B. Among them, the characteristic genera from GZ were *Delftia, Brachymonas, Bradyrhizobium, Pseudomonas, Acetobacterium*, and *Pelomonas*. The characteristic genera from HUB were *Comamonas, Cedecea, Sphingobacterium, Paenibacillus, Aquabacterium, Klebsiella*, and *Clostridium sensu stricto 10*. The characteristic genera from HUN were *Burkholderia-Caballeronia-Paraburkholderia, Thauera, Vagococcus, Blautia*, and *Bacteroides*. The characteristic genera from SX were *Chryseobacterium, Escherichia-Shigella*, and *Brevundimonas*.

### 3.6. Correlation analysis of EC dominant endophytic bacteria and active ingredients

The correlation between the top 20 dominant genera and active ingredients was analyzed using the Spearman correlation coefficient method, as shown in Figure 6C. Pinoresinol diglucoside had significant positive correlations with *Delftia* (*r* = 0.45, *P* < 0.01) and *Brevundimonas* (*r* = 0.32, *P* < 0.05). Geniposidic acid had significant positive correlations with *Sphingobacterium* (*r* = 0.45, *P* < 0.01), *Cedecea* (*r* = 0.35, *P* < 0.05), *Klebsiella* (*r* = 0.37, *P* < 0.05), and *Paenibacillus* (*r* = 0.32, *P* < 0.05) and significant negative correlations with *Pseudomonas* (*r* = −0.38, *P* < 0.05), *Pelomonas* (*r* = −0.38, *P* < 0.05), *Escherichia-Shigella* (*r* = −0.38, *P* < 0.05), *Sphingomonas* (*r* = −0.31, *P* < 0.05), and *Corynebacterium* (*r* = −0.32, *P* < 0.05). Geniposide had significant positive correlations with *Thauera* (*r* = 0.33, *P* < 0.05) and *Burkholderia-Caballeronia-Paraburkholderia* (*r* = 0.33, *P* < 0.05) and a significant negative correlation with *Klebsiella* (*r* = −0.37, *P* < 0.05). Syringaresinol diglucoside had a significant positive correlation with *Massilia* (*r* = 0.37, *P* < 0.05).

## 4. Discussion

Plants have long been an important part of natural therapies of the world, and there have been numerous examples of natural active ingredients extracted from medicinal plants to develop novel and effective preparations (Narayanan and Glick, 2022). In *E. ulmoides*, as an endemic economic tree species and source of tonic herbs in China, screening endophytes associated with active ingredients from its medicinal parts is an effective way to improve the quality of medicinal materials and meet the needs of pharmaceutical and agricultural production.

High-throughput sequencing technology has unique advantages in analyzing the characteristics of microbial communities. It can obtain larger and more comprehensive information on endophytic bacteria by directly extracting total DNA in the sample environment and sequencing, which is superior to the traditional culture methods (Glenn et al., 2019; Quijada et al., 2020). In previous studies, Li et al. (2007) isolated 122 strains of endophytic bacteria in EC by culturing, including 47 strains of endophytic bacteria. Ma et al. (2021) isolated EC endophytes by plate scribing and obtained 37 strains of endophytic bacteria, of which 21 strains were identified as belonging to the genera *Acinetobacter, Alcaligenes, Staphylococcus*, and *Bacillus*. In contrast, this study employed 16S rRNA sequencing to detect a significantly higher number of 1,149 genera in EC samples from GZ, HUB, HUN, and SX origins, highlighting the previously underestimated diversity and abundance of endophytic bacteria colonized in EC.

In general, the higher the abundance and diversity of the endophytic microbiota, the higher the metabolic stability and the material communication frequency of the microbiota (Cai et al., 2021). From the diversity results of alpha and beta, it could be seen that the endophytic bacteria diversity and richness of EC from HUN were the highest, and the difference was the largest with the other three origins. However, it is worth noting that the contents of the EC active ingredients from HUN were not outstanding, and only geniposide content was higher than other origins. In contrast, although the diversity and richness of EC endophytic bacteria from HUB were relatively low, the aucubin and geniposidic acid levels were the highest among the four origins. This means that the active ingredient contents in EC may be more influenced by the structural compositions of endophytic bacteria or certain specific bacteria but have little to do with the diversity and richness of species. This was also evidenced by the results of the correlation analysis, where the diversity indexes were not significantly related to the five active ingredient contents. Furthermore, the relationship between endophytic bacteria and active ingredient contents of EC was complex, and no single genus of bacteria simultaneously promoted or inhibited the contents of five active ingredients. For example, *Klebsiella* showed a significant positive correlation with geniposidic acid content, but a significant negative correlation with geniposide content. Some members of the genus *Klebsiella* (e.g., *Klebsiella pneumoniae*) have been reported to have the potential to convert geniposide to genipinine (Kawata et al., 1991). This suggests that there are interactions between different EC endophytic bacteria, which jointly affect the contents of active ingredients.

Endophytes have been studied for a century since De Barry introduced the term “endophytes” in 1866. However, research studies on endophytic fungi of medicinal plants have only been gaining attention in the last 30 years and even less on bacteria (Zheng et al., 2021; Anand et al., 2023). In fact, people have only a little understanding of endophytic bacteria's role in synthesizing iridoids and lignans in *E. ulmoides*. Furthermore, the current research into the origin and isolation of EC endophytic bacteria containing active ingredient synthesis gene clusters is still in its preliminary stages (Raimi and Adeleke, 2021). At the genus level, *Comamonas* was the dominant genus for the four EC origins, with the highest relative abundance in the HUB origin (20.71%) and the lowest in the HUN (3.02%), while it was also the characteristic bacterium of the HUB. *Comamonas* is a group of gram-negative bacteria that is abundant in a variety of environments including soil, plants, and water sources (Hem et al., 2022). Although *Comamonas* is a potentially pathogenic bacterium for humans, studies have shown the role of *Comamonas* in degrading exogenous pollutants and heavy metal contamination (Ryan et al., 2022). For instance, Wang et al. (2023) reported that the co-culture of *Comamonas* and *Enterobacter* reduced the accumulation of the heavy metal element Cd in rice. *Cedecea* is the second most abundant genus among all samples, and together with *Sphingobacterium, Paenibacillus*, and *Klebsiella*, these are the characteristic bacteria in the HUB group. These characteristic bacteria are significantly positively correlated with geniposidic acid. *Cedecea* has been found in the endophytic bacteria of medicinal plants such as *Rosmarinus officinalis* and Aloe vera (Akinsanya et al., 2015; Sharma et al., 2021; Ryan et al., 2022; Wang et al., 2023), but most reports on *Cedecea* have focused on its clinical pathogenicity (Ramaswamy et al., 2019; Thompson and Sharkady, 2021). *Sphingobacterium* is a gram-negative bacterium with wide distribution and strong stress resistance, and rarely causes human infection (Cai et al., 2020). *Paenibacillus* is the potential source of action for various antibacterial substances, and the chitinase and crystal protein secreted by it can effectively kill the larvae of beetle pests (Lorentz et al., 2006; Sharma et al., 2013; Grady et al., 2016). In addition, *Paenibacillus* plays an important role in promoting plant growth by facilitating phosphorus uptake, producing auxin, and inducing systemic resistance to pathogenic bacteria (Patten et al., 2013; Grady et al., 2016). Weak pathogenicity and positive effects on plant growth can be considered as one of the screening criteria for strains. Therefore, *Paenibacillu*s associated with the EC active ingredient content can be used as the characteristic bacterium from HUB for subsequent screening and verification. The same was true for the characteristic bacterium *Delftia* in GZ, which had a significant positive correlation with pinoresinol diglucoside. Previous studies have shown that *Delftia* has a good application prospect in promoting plant growth and bioremediation (Bhat et al., 2022). Some of its members can be used to reduce the accumulation of Cd elements, agricultural inoculants, or bioremediation agents (Bhat et al., 2022; Lo et al., 2022). In addition, there are other dominant endophytic bacteria including *Thauera, Massilia*, and *Sphingomonas* that have significant correlations with EC active ingredients. They have roles such as degrading aromatic compounds (Yang E. D. et al., 2019), producing polymers (Asaf et al., 2020), or secreting plant growth hormones (Liu et al., 2019).

This study provided initial insights into the relationships between EC endophytic bacteria of varying origins and active ingredients. Nevertheless, there are certain limitations that should be acknowledged, such as the limited sample size and analysis on the dominant genera only, which may cause the omission of some crucial endophytic bacteria.

## 5. Conclusion

This study demonstrates that the endophytic bacterial community of EC exhibits a diverse species composition, with significant differences observed among bacterial taxa from different origins. Meanwhile, certain endophytic bacterial genera, such as *Comamonas, Sphingobacterium*, and *Klebsiella*, are closely associated with the main active ingredients of EC. However, the mechanisms by which these endophytic bacteria influence the production of effective ingredients of EC remain largely unknown and warrant further research.

## Data availability statement

The datasets presented in this study can be found in online repositories. The names of the repository/repositories and accession number(s) can be found below: https://www.ncbi.nlm.nih.gov/, PRJNA933400.

## Author contributions

XL maintained and performed studies, wrote the manuscript, and revised the manuscript. DW and PL were mainly responsible for the determination of the active ingredients in the Eucommiae cortex and provided funding sources. KZ, JL, and XY reviewed the manuscript and participated in the analysis of the data. YP managed and coordinated the research activity planning and execution and provided financial support for the project leading to this publication. All authors contributed to the article, decided to submit the manuscript for publication, and approved the submitted version.
